# Possible effects of glimepiride beyond glycemic control in patients with type 2 diabetes: a preliminary report

**DOI:** 10.1186/1475-2840-13-15

**Published:** 2014-01-14

**Authors:** Ikuko Nakamura, Jun-ichi Oyama, Hiroshi Komoda, Aya Shiraki, Yoshiko Sakamoto, Isao Taguchi, Atsushi Hiwatashi, Aiko Komatsu, Masayoshi Takeuchi, Sho-ichi Yamagishi, Teruo Inoue, Koichi Node

**Affiliations:** 1 Department of Cardiovascular Medicine, Saga University, Saga 849-8501, Japan; 2 Department of Cardiovascular Medicine, Dokkyo Medical University, Mibu, Tochigi, Japan; 3 Department of Advanced Medicine, Medical Research Institute, Kanazawa Medical University, Uchinada, Ishikawa, Japan; 4 Department of Pathophysiology and Therapeutics of Diabetic Vascular Complications, Kurume University School of Medicine, Kurume, Fukuoka, Japan

**Keywords:** Glimepiride, Advanced glycation end products, Cytokines/chemokines, Growth factors, Diabetes mellitus type 2

## Abstract

**Background:**

The purpose of this study was to elucidate the effects of glimepiride on the levels of biomarkers related to cardiovascular regulation in patients with type 2 diabetes mellitus.

**Methods and results:**

Thirty-four patients with type 2 diabetes received glimepiride for 24 weeks. Significant decreases in the levels of glyceraldehyde-derived advanced glycation end products, (glycer-AGE: toxic AGE), eotaxin and fibroblast growth factor (FGF)-2 were recognized after the administration of glimepiride. Moreover, there were trends for there to be increases in the levels of granulocyte-colony stimulating factor (G-CSF) and granulocyte macrophage-colony stimulating factor (GM-CSF), and decreases in the levels of fractalkine, soluble CD40 ligand (sCD40L), macrophage inflammatory protein (MIP)-β, vascular endothelial growth factor (VEGF) and soluble receptor for AGE (sRAGE).

**Conclusions:**

Glimepiride may have potent anti-oxidative, anti-inflammatory and angiogenic properties and it may potentially repair tissue damage by decreasing the levels of toxic AGE and increasing colony-stimulating factors.

## Introduction

Diabetes mellitus continues to increase in terms of the number of affected and in significance worldwide, and is a growing burden with regard to public health. It is estimated that there were 285 million people worldwide with diabetes in 2010, and this number is expected to increase 439 million by 2030 [1]. Type 2 diabetes is a metabolic disorder characterized by chronic hyperglycemia resulting from a progressive insulin secretory defect on the background of insulin resistance, usually leading to absolute insulin deficiency, which results in complex phenomena exacerbated by central obesity [2], and increases the risk for atherosclerosis and related cardiovascular disease [3-6]. Therefore, optimal anti-diabetic treatment requires beneficial effects that can help to prevent diabetic complications, in addition to providing good glycemic control.

Glimepiride is a second-generation sulfonylurea that stimulates pancreatic β cells to release insulin. This agent mainly stimulates insulin secretion, but has also been shown to have additional extra-pancreatic effects in animal models [7,8]. The aim of this study was to elucidate the beneficial effects of glimepiride on cardiovascular system-related biomarkers in diabetic patients.

## Methods

### Subjects

Forty-five patients who agreed to participate in this study were enrolled. The entry criteria included 1) age > 30 years old, 2) type 2 diabetes with a hemoglobin A1c (HbA1c) value > 6.5% and 3) under treatment with diet, exercise, alpha glucosidase inhibitors (α-GIs), and/or first generation sulfonylurea drugs, such as glibenclamide (< 5 mg/day) or gliclazide (< 80 mg). The exclusion criteria were 1) severe diabetic complications such as > stage 3A nephropathy or > stage B retinopathy, 2) liver dysfunction, as indicated by an AST > 80 IU/l or ALT > 80 IU/l, 3) cancer, 4) renal dysfunction (serum creatinine > 2.0 mg/dl) and 5) receiving the treatment with oral glimepiride, metformin or pioglitazone. The study protocol was approved by the Institutional Review Committee on Human Research at Saga University and by other institutions. Informed consent was obtained from each patient.

### Study protocol

All patients received treatment with glimepiride after study entry. Glimepiride was started as: 1) a new medication in diabetic patients receiving diet/exercise therapy but no anti-diabetic agents; 2) additional therapy in combination with α-GIs in patients with poorly controlled glucose; or 3) in exchange for first generation sulfonylurea agents, such as glibenclamide or gliclazide in patients with poorly controlled glucose. The dose of glimepiride was started at 1 mg daily and increased up to 6 mg daily until a value of HbA1c < 6.5% was achieved in patients who received glimepiride as a new medication or an additional therapy to α-GIs. If the glimepiride was given in place of glibenclamide or gliclazide, the starting dose of glimepiride was decided by referring to previous reports indicating that 1 mg of glimepiride corresponded to 1.5 mg of glibenclamide or 20 mg of gliclazide. Adverse events were recorded continuously. In all of the entry patients, various blood biomarkers related to cardiovascular pathophysiology were measured at baseline before starting glimepiride treatment and 24 weeks after the start of glimepiride treatment.

### Measurement of advanced glycation end products

The concentrations of glyceraldehyde-derived advanced glycation end products (glycer-AGE), one of the toxic AGE present in the serum, were measured with a competitive ELISA using an immunopurified glycer-AGE antibody [9]. In brief, 96-well microtiter plates were coated with 1 μg/ml glycer-AGE-bovine serum albumin (BSA) per well, and were kept overnight in a cold room. The wells were washed three times with 0.3 ml of phosphate-buffered saline (PBS)-Tween-20. Wells were then blocked by incubation for 1 h with 0.2 ml of PBS containing 1% BSA. After washing with PBS-Tween-20, test samples (50 μl) were added to each well as a competitor for 50 μl of the glycer-AGE antibody (1:1000), followed by incubation for 2 h at room temperature with gentle shaking by a horizontal rotary shaker. The wells were then washed with PBS-Tween-20 and developed with an alkaline-phosphatase-linked anti-rabbit IgG utilizing *p*-nitrophenyl phosphate as a colorimetric substrate. The results are expressed as glycer-AGE units (U) per milliliter of serum, with 1 U corresponding to 1 μg of glycer-AGE-BSA standard. The sensitivity and intra- and inter-assay coefficients of variation were 0.01 U/ml, 6.2 and 8.8%, respectively. The level of the soluble form of the receptor for AGE (sRAGE) was measured using an ELISA kit (R & D Systems Inc, Minneapolis, MN, USA), as described previously [10].

### Other measurements

The following parameters were assessed at baseline before starting glimepiride treatment and 24 weeks after starting the glimepiride treatment. The fasting plasma levels of glucose and insulin and the hemoglobin A1c (HbA1c) were measured, and the insulin resistance was determined using Matthews’s homeostasis model assessment (HOMA). The HOMA-β and HOMA-R were based on the following formula: 360 × fasting plasma insulin (μU/mL)/[fasting blood glucose (mg/dL) – 63] and fasting blood glucose (mg/dL) × fasting plasma insulin (μU/mL)/405, respectively. We measured the brain natriuretic peptide (BNP) level using a chemiluminescent enzyme immunoassay method. High-sensitivity C-reactive protein (hsCRP) was measured by particle-enhanced immunonephelometric assays. High molecular weight (HMW) adiponectin was measured using a sandwich ELISA kit (Fujirebio, Tokyo, Japan) based on detection by a monoclonal antibody against human HMW adiponectin, IH7 [11]. The plasma PTX3 levels were measured with a sandwich ELISA based on a previously described method [12]. Then, we performed a fluorescent microbead-based assay to measure multiple cytokines and chemokines using a Luminex 100 instrument (Luminex Corp, Austin, TX) with a MILLIPLEX MAP Human Cytokine/Chemokine kit (Millipore, Billerica, MA), as described previously [13], to determine the levels of various cytokines, chemokines and growth factors, including interleukin (IL)-1β, IL-6, tumor necrosis factor (TNF)-α, monocyte chemoattractant protein (MCP)-1, interferon (IF)-γ, granulocyte-colony stimulating factor (G-CSF), granulocyte macrophage-colony stimulating factor (GM-CSF), eotaxin, fibroblast growth factor (FGF)-2, vascular endothelial growth factor (VEGF), soluble CD40 ligand, fractalkine and macrophage inflammatory protein (MIP)-β.

### Statistical analysis

The data were expressed as the means ± SEM unless otherwise indicated. The statistical significance of differences between groups were analyzed by Student’s *t*-test or the Wilcoxon signed-ranks test using the SPSS software program, and values of p < 0.05 were considered to be significant.

## Results

### Baseline characteristics of the patients

Of the 45 patients enrolled in this study, six (13%) were taking glibenclamide, one (2%) was taking gliclazide and fourteen (31%) were taking α-GI at the time of entry. All 45 patients were taking antihypertensive medications, such as angiotensin-converting converting enzyme inhibitors (ACEI) and/or angiotensin II receptor blockers (ARB) (27 patients, 60%), β-blockers (15 patients, 33%) or calcium channel antagonists (30 patients, 67%). Nine (20%) patients were taking diuretics and 26 (57%) patients were taking hydroxymethylglutaryl-CoA (HMG-CoA) reductase inhibitors. The patients had histories of the following cardiovascular diseases: old myocardial infarction in 10 (22%), angina pectoris in seven (16%), old cerebral infarction in three (7%), heart failure in three (7%), abdominal aortic aneurysm in two (4%), paroxysmal atrial fibrillation in two (4%), transient ischemic attack in one (2%) and an aortic dissection in one (%) patient.

**Figure 1:**
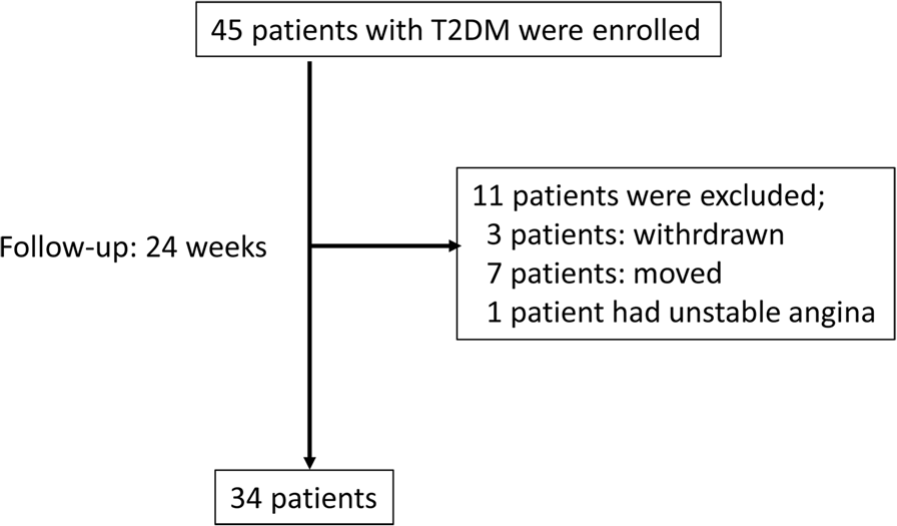
Participant flow.

Of the 45 enrolled patients, 11 patients were excluded from the analysis because of failed follow-up in 10 patients (3 patients: withdrawn, 7 patients: moved) and unstable angina pectoris in one patient, and thus, a total of 34 patients completed the study protocol (Figure 1). The clinical profiles of the 34 evaluated patients are shown in Table 1.

**Table 1:** Baseline characteristics of the patients

		**n (%)**
Gender	Male	24 (70.6%)
	Female	10 (29.4%)
Age (y.o.)		64.7 ± 1.4
Height (cm)		160.6 ± 1.5
Body weight (kg)		66.5 ± 1.9
BMI		25.7 ± 0.7
Complication(s)	Retinopathy	0%
	Nephropathy	2 (5.90%)
	Neuropathy	0%
	Hypertension	26 (76.5%)
	Dyslipidemia	17 (50.0%)
	CAD or CVD	11 (32.4%)
Drug(s)	ACEI/ARB	21 (61.8%)
	Ca antagonist	24 (70.6%)
	Diuretics	9 (26.5%)
	HMG-CoA reductase	22 (64.7%)

BMI: body mass index, CAD: coronary artery disease, CVD: cerebrovascular disease, ACEI: angiotensin converting enzyme inhibitor, ARB: angiotensin 2 receptor blocker, HMG-CoA reductase: hydroxymethylglutaryl-CoA reductase.

### Glycemic control

Figure 2.

**Figure 2:**
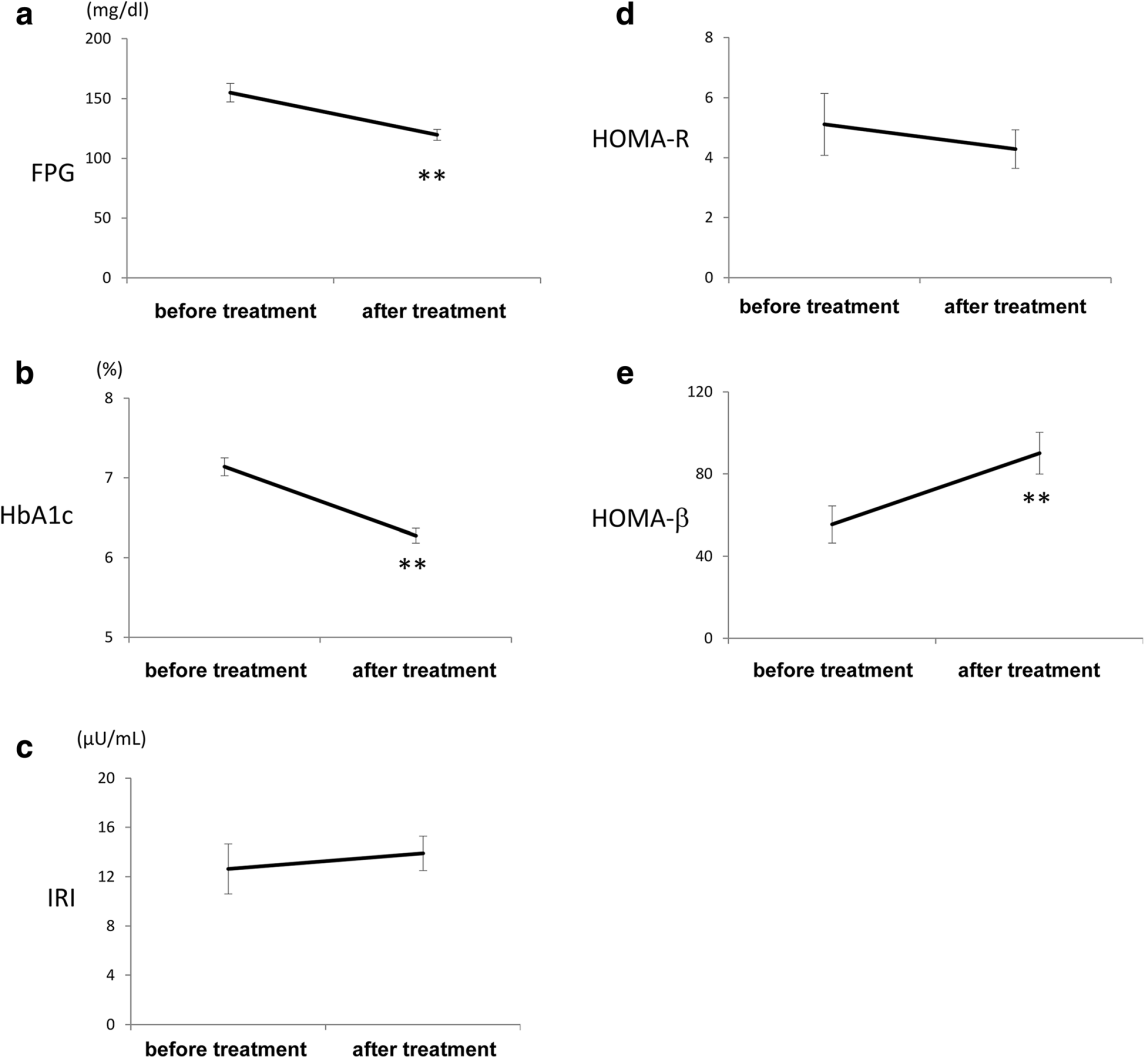
**The changes in the levels of fasting plasma glucose (a), HbA1c (b), fasting plasma insulin (c), HOMA-R (d) and HOMA-β (e) after the treatment with glimepiride.** *p < 0.05, **p < 0.01 versus before treatment. HbA1c = hemoglobin A1c, HOMA = homeostasis model assessment.

The levels of HbA1c and fasting plasma glucose (FPG) decreased, and the HOMA-β value increased significantly following the start of glimepiride. However, the plasma insulin level and HOMA-R value were comparable before and after the treatment (Figure 1). After 24 weeks, 23 patients achieved a HbA1c < 6.5%. At 24 weeks, the mean dose of glimepiride was 2.6 mg, and 13 patients (38%) who received treatment with only 1 mg of glimepiride achieved a HbA1c < 6.5%. Six patients (17%) had mild hypoglycemia who had symptoms but recovered without intensive assistance and no patient had severe hypoglycemia who required a third party to intervene actively during the study.

### Assessment of biomarker changes after the administration of glimepiride

**Figure 3:**
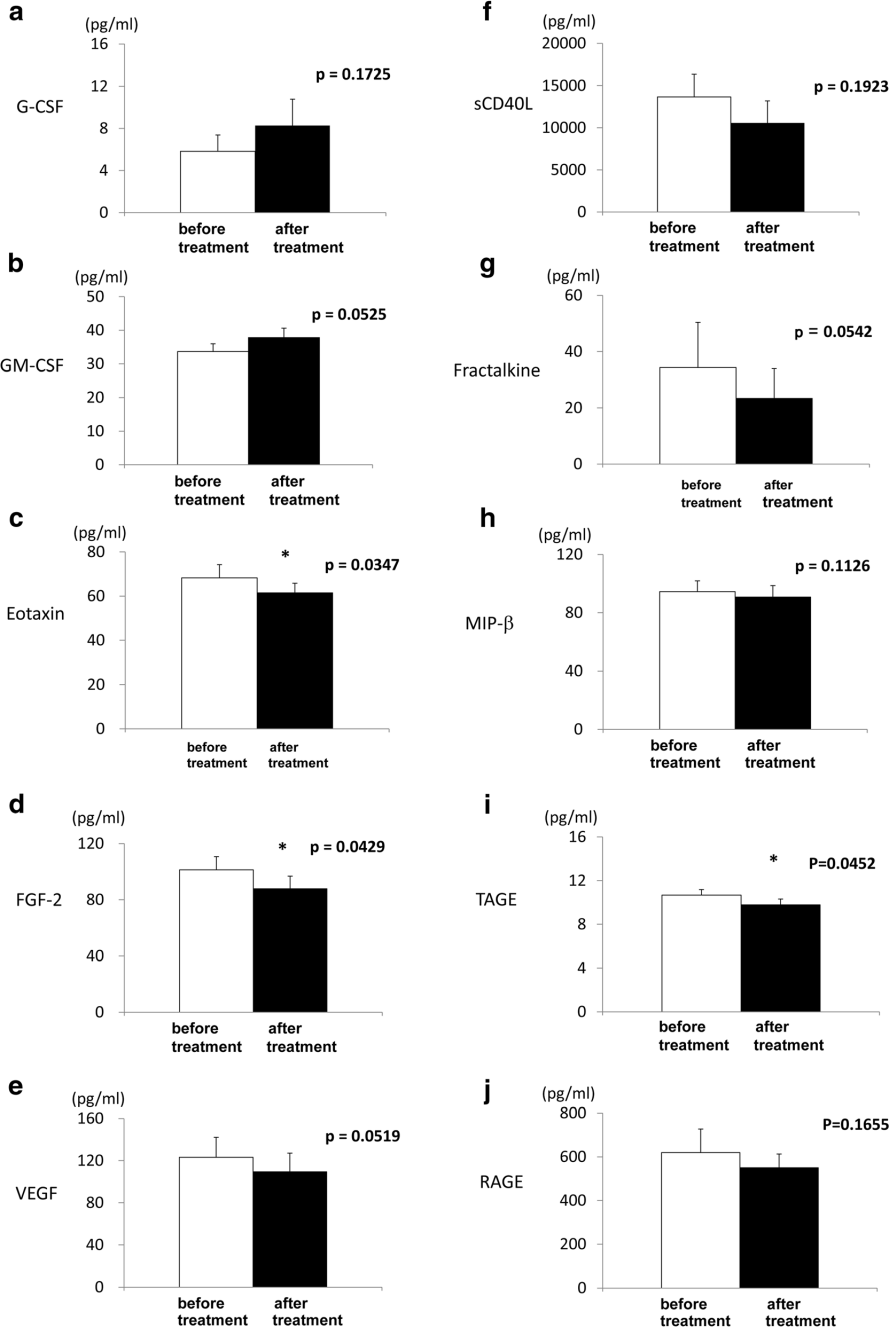
**The changes in the serum or plasma levels of G-CSF (a), GM-CSF (b), eotaxin (c), FGF-2 (d), VEGF (e), soluble CD40 ligand (f), fractalkine (g), MIP-β (h), glycer-AGEs (i) and sRAGE (j) by the treatment with glimepiride.** Open bars indicate "before treatment" and solid bars indicate "after treatment". *p < 0.05 versus before treatment. G-CSF = granulocyte colony-stimulating factor, GM-CSF = granulocyte macrophage-colony stimulating factor, FGF = fibroblast growth factor, VEGF = vascular endothelial growth factor, MIP = macrophage inflammatory protein, glycer-AGE = glyceraldehyde-derived advanced glycation end products, sRAGE = soluble receptor for advanced glycation end products.

The levels of hsCRP, PTX3 and most of the inflammatory cytokines, including IL-1β, IL-6, TNF-α, IFN-γ and MCP-1, did not change after glimepiride treatment, while the BNP level significantly decreased (Table 2). The levels of eotaxin (p = 0.0347), FGF-2 (p = 0.0429) and toxic AGE (p = 0.0452) decreased significantly after the treatment. Moreover, a trend toward an increase in the levels of G-CSF (p = 0.1725) and GM-CSF (p = 0.0525), and a decrease in the levels of VEGF (p = 0.0519), soluble CD40 ligand (p = 0.1923), fractalkine (p = 0.0542), MIP-β (p = 0.1126) and soluble RAGE (p = 0.1655) were observed (Figure 3).

**Table 2:** The effects of the changes in biochemical markers on DM patients

	**Before administration**	**24 weeks after treatment**	**p**
BNP	39.2 ± 10.8	33.6 ± 10.4*	0.048
hsCRP	1859.5 ±426.7	2600.2 ± 745.9	0.254
HMW adiponectin	3.58 ± 0.43	3.82 ±0.53	0.468
PTX3	2.39 ± 0.26	2.16 ±0.29	0.082
IL-1β	2.23 ± 7.66	2.57 ±8.91	0.297
IL-6	3.62 ± 8.90	3.69 ±9.18	0.879
TNF-α	4.34 ±2.00	4.38 ± 1.17	0.872
MCP-1	554.78 ±204.55	565.88 ± 211.85	0.581
IFN-γ	3.94 ± 5.52	3.97 ± 4.75	0.9419

BNP: brain natriuretic peptide, hs CRP: high-sensitive C-reactive protein, HMW: high molecular weight, PTX3 pentraxin-3, IL: interleukin, MCP-1: monocyte chemoattractant protein-1, IFN: interferon.

*p<0.05 vs before treatment.

## Discussion

In this study, we found that glimepiride significantly suppressed the plasma levels of eotaxin, FGF-2 and toxic AGE, and showed a trend to reduce those of fractalkine, sCD40L, MIP-β, VEGF and sRAGE, and to increase the levels of G-CSF and GM-CSF.

Sulfonylureas may increase the risk of cardiovascular events in patients with type 2 diabetes [14]. However, glimepiride seems to have fewer unfavorable cardiovascular effects compared to other sulfonylureas. Glimepiride upregulated eNOS activity and inhibit NF-kB activation in human umbilical vein endothelial cells [15]. In open-chest dogs, intracoronary infusion of glibenclamide, gliclazide and glimepiride all reduced coronary blood flow, increased coronary resistance, depressed the mechanical activity of the heart, enhanced myocardial O2-extraction, reduced the serum potassium level and induced a moderate endocardial ST-segment elevation, but glimepiride to a significantly less extent than glibenclamide and gliclazide [16]. Likely underlying the favorable cardiovascular effects of glimepiride are its induction of ischemic preconditioning, suppression of ventricular tachycardia and lowering of the blood pressure, unlike other sulfonylureas [17-19]. Unfortunately, these effects has previously been shown only in animal experiments. Therefore, in this study, we provided the first demonstration of the clinical effects of glimepiride in humans. Previous reports revealed that glimepiride and rosiglitazon reduced the levels of hsCRP, AGEs [20], lipoprotein, homocyctein and plasminogen activator inhibitor-1 [21] increased adiponectin [22] in T2DM. Recently phase 3 trial of empagliflozin, SGLT2 inhibitor, versus glimepiride as add-on to metformin in T2DM patients are being performed [23]. However, the study about exenatide, GLP-1 agonist, versus glimepiride added to metformin revealed that glimepiride reduced hsCRP and increased adiponectin although exenatide did with great extent [24]. Moreover, glimepiride could not prevent the atherosclerosis of carotid intima-media thickness [25] and coronary atherosclerosis [26] compared with pioglitazone.

AGEs are oxidative products formed from the reaction between carbohydrates and a free amino group of proteins, which are provoked by reactive species and are greatly accelerated in response to the hyperglycemia and oxidative stress that occur in diabetic subjects [27]. Since the AGEs activate nuclear factor-κB (NF-κB) and activator protein-1 (AP-1), transcription factors, which upregulates the expression of genes involved in vascular injury and endothelial dysfunction, in microvascular endothelial cells and induce angiogenesis, lowing the AGEs may contribute to ameliorating diabetic vasculopathy [20,28-30]. The serum levels of toxic AGE were inversely associated with the number of endothelial progenitor cells in apparently healthy subjects [31]. The reduction in toxic AGE and sRAGE by glimepiride in our present study, therefore, the results may suggest that glimepiride have a potential benefit for repairing the vascular injury and stop angiogenesis in diabetic patients.

In this study, there was a trend toward an increase in the level of G-CSF and GM-CSF after the administration of glimepiride for 24 weeks, suggesting that glimepiride has effects on angiogenesis, which is a pivotal mechanism that influences several physiological and pathological processes, including wound healing, because GM-CSF is known to induce bone marrow precursors to protect against the development of diabetes and to induce wound healing in diabetic mice [32-34]. In our study, the plasma levels of FGF-2 and VEGF were also reduced after the administration of glimepiride. FGF-2 is a potent mitogen in endothelial cells and smooth muscle cells that is released after endothelial injury [35] and is capable of inducing smooth muscle cell migration and proliferation, leading to neointima formation [36,37]. The plasma FGF-2 level is low or undetectable in healthy subjects [38], but increases in microalbuminuric adult type 2 diabetic patients.

Both FGF-2 and VEGF are considered to be key factors in the angiogenic response [39,40], and induce increased vascular permeability. The vascular hyperpermeability and increased blood flow caused by elevated tissue glucose and sorbitol levels can be blocked by neutralizing monoclonal and polyclonal antibodies directed against VEGF [41], suggesting that a sorbitol pathway-linked increase in VEGF may be involved in the hemodynamic changes and loss of endothelial cell barrier integrity induced by diabetes. Increased glucose induces the production of VEGF and FGF-2 to promote angiogenesis, and to protect against tissue injury. In our study, however, glimepiride reduced the plasma levels of VEGF and FGF-2.

We believe that the results of treatment may depend on the decreased induction of cytokines resulting from the glycemic improvement or via the pleiotropic effects of the drug. It was previously reported that patients with type 1 diabetes showed higher concentrations of plasma GM-CSF, soluble CD40, soluble CD40 ligand, MIP-β [42], and that the acute effect of clamped hyperglycemia increased the urinary excretion of eotaxin, FGF-2, GM-CSF, TNF-α and soluble CD40 ligand in patients with type 1 diabetes. Based on these results, our data supplements the favorable cardiovascular effects of glimepiride, which is in contrast to the unfavorable cardiac effects of other sulfonylureas.

In our study, the level of eotaxin also decreased after glimepiride treatment. Eotaxin is a potent eosinophil chemoattractant that is a member of the CC chemokine subfamily of inflammatory and immunoregulatory cytokines. Although the role of eotaxin in diabetes is still being elucidates, it is thought to be substantially involved in the development of the disease in patients with type 1 diabetes [43]. Moreover, in patients with type 2 diabetes, insulin infusion reduced the levels of eotaxin and MCP-1 [44], and the eosinophil count is also related to albumin excretion in males [45]. Although the role of eotaxin in diabetes is still unclear, the protein sequence of human eotaxin is 66% identical to that of human MCP-1. Therefore, the decrease in eotaxin by glimepiride may reflect its potent anti-diabetic properties.

### Limitations

This was a preliminary single-arm study that was designed to evaluate only six months of treatment in a small number of subjects. Glimepiride is already widely used anti-diabetic drug and it is hard to avoid the use of glimepiride in the control group for 6 months in real clinical world when randomized placebo-control group is performed. Therefore, we performed a single-arm study as preliminary report.

In this study, we confirmed that glimepiride may have angiogenic properties. However, we did not evaluate the effects of the treatment on diabetic retinopathy because there were no patients with diabetic retinopathy in our group. Therefore, future studies will be needed to determine whether glimepiride can ameliorate or prevent diabetic complications including macro- and microagiopathies.

We also did not elucidate whether these phenomenona depended specifically on the glycemic control or were affected by other feature of glimepiride. However, we confirmed that these results did not correlate with the lowering of the blood glucose level or the level of HbA1c. Therefore, these results may depend on the unique features of the drug itself.

## Conclusions

In conclusion, the present study demonstrated that glimepiride may have beneficial effects beyond improving the glycemic control by reducing the toxic AGE and inducing CSFs.

## Competing interest

NK was granted by scholarship found from Sanofi-aventis K.K.

## Authors’ contributions

IN acted as "clinical investigator". She carried out the physical examinations, taking blood samples and drafted the manuscript. JO was involved in managing and planning the project, and edited and revised manuscript. HK and AS measured the levels of biomarkers in blood sampling. AK, AH and YS recruited the patients and carried out physical examinations. KN acted as "senior investigator" planning and observing the project. MT and SY carried out the measurement of RAGE from the blood sampling. IT and TI advised the design and performance of this study. All authors read and approved the final manuscript.
